# What are the priority-setting approaches for HIV/AIDS, TB and malaria programmes in Ghana? A qualitative perspective from key informants

**DOI:** 10.1136/bmjph-2024-001097

**Published:** 2024-12-12

**Authors:** Genevieve Cecilia Aryeetey, Augustina Koduah, Adjeiwa Akosua Affram, Richmond Owusu, Francis Ruiz, Anna Vassall, Justice Nonvignon

**Affiliations:** 1 Health Policy, Planning and Management, University of Ghana School Public Health, Accra, Ghana; 2 Pharmacy Practice and Clinical Pharmacy, University of Ghana School of Pharmacy, Accra, Ghana; 3 Global Health and Development, London School of Hygiene & Tropical Medicine, London, UK; 4 Health Financing and Economics, World Health Organization, Geneva, Switzerland

**Keywords:** HIV, Public Health, Acquired Immunodeficiency Syndrome

## Abstract

**Introduction:**

Worldwide, countries have the challenge of meeting the ever-increasing demand for healthcare amidst limited resources. While priority setting is necessary in all settings, it is especially critical in low- and middle-income countries because of their often-low budgetary allocations for health. Despite the long history of disease programmes supported by the Global Fund to Fight AIDS, Tuberculosis and Malaria (GFATM) in Ghana, there is limited evidence on the approaches used in priority setting for the three disease programmes. This study aimed at exploring the priority-setting approaches adopted by the GFATM-supported programmes in Ghana.

**Methods:**

In-depth interviews of ten key informants from the three disease programmes, the Ministry of Health and global health partners were conducted. Interviews were transcribed verbatim and analysed both inductively and deductively.

**Results:**

We identified four main approaches for priority setting: (1) identification of health needs, (2) stakeholder participation, (3) transparency of the process and (4) contextual factors. Priorities were identified through national health strategies and mandates, development/health partners and global mandates and internally generated data and surveillance. The main actors participating in the decision-making or priority setting were ministries and agencies, development partners, research institutions, committees and working groups. These actors had varying influences and power. The involvement of the general public was limited in the priority-setting process. The approaches were often documented and disseminated through various mediums. Contextual factors reported were mainly barriers that affected priority setting, and these included inadequate funding, aligning priorities with funders and interruptions in the priority-setting process.

**Conclusion:**

While explicit priority-setting approaches are being expanded globally to support resource allocation decisions in health more generally, evidence from our study suggests that their use in the three GFATM-supported programmes was limited.

**WHAT IS ALREADY KNOWN ON THIS TOPIC**

- Many studies identify and recommend explicit approaches to priority setting. These approaches have been demonstrated as a transparent and systematic approach for effective resource allocation. These explicit approaches are however only part of the priority-setting process. It is at the discretion of governments, institutions and organisations to choose how to carry out their preferred explicit priority-setting approach, including if and how they use a framework to guide the process.

**WHAT THIS STUDY ADDS**

- There appears to be limited application of explicit approaches to priority setting in the three Global Fund to Fight AIDS, Tuberculosis and Malaria (GFATM)-supported programmes in Ghana. Approaches for priority setting followed similar frameworks identified in the literature; however, there were gaps in their implementation for successful priority setting.

**HOW THIS STUDY MIGHT AFFECT RESEARCH, PRACTICE OR POLICY**

- As low- and middle-income countries face a higher burden of disease and limited resources, priority setting will be central to achieving universal health coverage. Ensuring explicit approaches guide priority setting is particularly important as the GFATM looks to effectively allocate finite financial resources amid global economic insecurities.

## Introduction

 Priority setting is the process of making decisions about how best to allocate limited resources to improve population health.^1,2^ Priority setting is complex. It involves diverse stakeholders with varying interests and influences.^3^ Priority setting and resource allocation have in the past often been implicit and politically driven. However, the current trend is to focus on the use of evidence, transparency and stakeholder engagement to inform decisions on appropriate health interventions that meet population health needs.^4^

While priority setting is necessary in all settings, it is especially critical in low- and middle-income countries (LMICs) because of their often-low budgetary allocations for health. Despite this urgency, resource allocation and decisions in LMICs regarding health service coverage and choice of intervention are often done in an ad hoc and non-transparent manner.^5^ In many LMICs, there are no institutional mechanisms for priority setting, leaving key decision-making on resource allocation to interest groups.^5,6^ This may lead to resource allocation decisions that may not result in optimum health system gains and outcomes.^5,7^

To date there is no single approach to priority setting. Several approaches have been used over the years, largely depending on the context. Menon *et al*^8^ described priority setting in four steps: (1) identification of healthcare needs, (2) allocation of resources, (3) communication of decisions to stakeholders and (4) management of feedback from them. Sibbald *et al*^9^ and Tomlinson^10^ explained that the outcomes of priority setting are likely to be achieved when stakeholder engagement, transparent decision-making process, information availability and management, consideration of country and institutional context and values are appropriately considered through the decision-making process.

Shiffman and Smith’s^11^ framework includes attending to and addressing (1) the power of actors, (2) the influence of ideas, (3) the nature of political contexts and (4) characteristics of the issue itself. Walton *et al*^12^ and Martin^13^ outlined that the process should be documented and transparent as well as fair and legitimate. It must also involve a wide range of stakeholders through stakeholder engagements and have a mechanism to address appeals and revisions through feedback. Priority setting also requires leadership to be effective.

Health priorities can also be set using explicit tools and approaches like health technology assessment (HTA), cost-effectiveness analysis and multiple criteria decision analysis (MCDA).^14,15^ These approaches emphasise their capacity for effective priority setting through the use of rigorous instruments and transparent analytic process to respond to diverse stakeholder needs.^16,17^ Their use appears limited in LMICs with studies implicating data quality and availability, health system context, limited human resource and financial capacity for their low uptake.^17,18^

Within the Ghanaian health system, efforts are being made to ensure efficiency, particularly mapping outcomes to the use of resources in service delivery. Over the past years, the Ministry of Health (MoH) together with its stakeholders set health priorities through the annual health summits and review processes.^19^

Malaria, HIV/AIDS and TB remain a major public health issue in Ghana despite major success in decreasing infections and mortality.^20–23^ There is a long history of malaria, TB and HIV/AIDS disease programmes supported by the Global Fund to Fight AIDS, Tuberculosis and Malaria (GFATM).^24^ However, there is limited evidence on the approaches used for priority setting. An inquiry into the priority-setting approaches of the GFATM-supported programmes in Ghana is particularly important as the programme looks to effectively allocate finite financial resources amid global economic insecurities.^25,26^ This study explored the approaches used for priority setting in the three national disease control programmes (HIV/AIDS, TB and malaria programmes) in Ghana which are supported by the GFATM.

## Methods

### Study design

We employed a qualitative design for this study, specifically key informant interviews.

### Research setting

The health system structure in Ghana, although decentralised, is still primarily top-down.^27^ While many groups are involved in the decision-making process, final decisions are taken by the central government.^28,29^ The Ghana Health Service is the implementing agency for the MoH. Various departments under the Ghana Health Service coordinate the implementation of health policies set by the MoH. These include The National AIDS Control Programme, The National Malaria Elimination Programme and the National Tuberculosis Control Programme under the directorate of Public Health of the Ghana Health Service.^30^ We conducted this research in the Greater Accra Region where the National offices of the three disease programmes are located.

### Sampling

Many participants (n=9) were sampled purposively following an eligibility criterion developed by the project team, including organisation, role (level and expertise) and experience (a year and above in service). One individual was sampled via snowballing through recommendation from an interviewee. Participants were selected to represent institutional knowledge of the approaches for priority setting of the three disease programmes at the central level. We did not include participants from other groups like civil society organisations and health workers because of their limited accessibility and participation in decision-making at the highest level.

Our participants included eight men and two women occupying positions in the MoH, the three National disease programmes, the GFATM and the WHO. Global health partners from the GFATM and the WHO were included in the study because the Global Fund supports the funding of these three disease programmes and the WHO provides technical support at the central level for the three disease programmes. We did not have control over accounting for gender because the individuals meeting our criteria were mostly males. Table 1 contains participant details.

**Table 1:** Characteristics of key informants

ID	Sex	Years of service	Programme/institution
**1**	M	13 (7)*	Malaria
**2**	M	6	Malaria
**3**	M	15 (6)*	HIV/AIDS
**4**	M	23 (5)*	TB
**5**	M	27	TB
**6**	M	13	HIV/AIDS
**7**	F	6	HIV/AIDS
**8**	F	3	WHO
**9**	M	n/a	Global Fund
**10**	M	1	MOH

* Years in current position.

### Ethics

Ethical approval was received from the College of Health Sciences Ethics and Protocol Review Committee (IRB:00006220).

### Data collection

The interviews were conducted between October and December 2021 by a trained research assistant. All interviews were conducted with an interview guide developed by the authors. The guide was developed by reviewing existing literature on priority setting as well as other qualitative studies conducted on priority setting. The authors met multiple times in-person and online to review and revise the guide to its final stage. Please see online supplemental appendix 1 for a sample of the question guide.

Potential participants were first contacted through emails with a letter introducing the study and requesting an interview. We reached saturation after 10 participants. While 10 individuals may be considered a relatively small number for a qualitative study, it is not an inadequate number to reach data sufficiency.^31,32^ Interviews were conducted virtually (n=6) and in-person (n=4). All participants provided verbal consent. All interviews were recorded. The participants interviewed were not involved in the design, conduct, reporting or dissemination of our study.

### Data analysis

The interviews were transcribed verbatim and stored in an encrypted folder only available and accessible to the authors. Data were analysed both inductively and deductively guided by existing frameworks for priority setting.^6,8–13,31,32^ Specifically, we looked for responses that mapped onto: (1) stakeholder participation to capture stakeholder engagement, communication and power, (2) transparency of the process to capture the information availability as well as fairness of the process and (3) contextual factors to capture leadership, nature of the political context and reflection of public values.

We adopted an inductive approach to generate original findings from the raw data within and outside of these frameworks. Inductive analysis was conducted following the structure for conducting thematic analyses by Braun and Clarke.^33^ This involved: (1) familiarisation with the data; (2) systematic data coding; (3) generating initial themes; (4) developing and reviewing themes; (5) refining, defining and naming themes; and (6) writing the report.

## Results

We identified four main themes which are (1) identification of health needs, (2) stakeholder participation (3) transparency of the process and (4) contextual factors. Under each theme, the sub-themes identified are presented below. We discovered that the approaches to priority setting are not mutually exclusive, and similarities may be evident in each approach. Please see online supplemental appendix 2 for the summary of the results.

### Identification of health needs

This theme relates to participants’ description of the approaches used to identify, which health issues to prioritise. Health needs were identified through (1) National health strategies and mandates, (2) development/health partners and global mandates and (3) internally generated data and surveillance.

#### National health strategies and mandates

According to some key informants, deciding health issues of priority was dependent on existing strategies and mandates set at the national level. In such instances, institutions had to align their goals and tasks with already established priorities.

… major decisions that we make are also part of the main policy for Ghana Health Service, because that’s where we also derive our mandates… you will also have to develop guidelines, policies that are connected to the general policy of the Ministry [of health], or Ghana Health Service (Respondent 5, TB Control program)

In other instances, health needs that are prioritised may not be set by the MoH and its affiliates. Rather, reflects wider political agendas.

I mean it depends on what is on the table. Either it is political agenda from the government…[sometimes] government also has its own beliefs that once its controlled from the Presidency then things can get done quickly…in this case the ministry will just be providing some technical activities and support. (Respondent 10, Ministry of Health).

#### Development/health partners and global mandates

In some cases, the mandates of development and health partners as well as guidelines set at the global level influenced priorities.

We are part of the global picture of HIV. So, if globally there is a target set, how do we buy in into it as well-- we also look at what is happening locally…. what are the gaps locally that will prevent us from achieving the global targets that have been set for all of us. (Respondent 7, National AIDS and STI Control Program)

#### Internally generated data and surveillance

The identification of health needs, in some cases, was informed by data and surveillance.

We collect continuously feedback on people’s experiences on treatment, for instance, and that informs any revisions in our guidelines. So apart from the global data and best practice review of this, we use routine work because we undertake monitoring and evaluation. (Respondent 3, National AIDS and STI Control Program)

### Stakeholder participation

This theme describes the role of actors and stakeholders in setting health priority for the three disease programmes. The sub-themes identified were: (1) stakeholder engagement, (2) revision of priorities and (3) power and influence.

#### Stakeholder engagement

A diverse range of stakeholders including individuals from committees and working groups, research institutions, development partners as well as the MoH and its agencies were engaged. As one participant described the process saying: ‘*It’s more of consultative approach…the document that we put together we invited all the stakeholders’ (Respondent 10, Ministry of Health*).

Despite the narrative of a collaborative engagement process involving multiple stakeholders, many of the stakeholders mentioned by participants were individuals who had worked within the health sector or academic setting. Only one stakeholder mentioned the inclusion of individuals for whom the programmes are intended (the end users), as well as the involvement of civil society organisations.

We work with all other stakeholders within the service, the ministry. And then outside the service; development partners and viewers, civil society to finalize or craft the policies and the interventions together… We take into consideration the need interest of our beneficiaries, the people living with HIV…. In fact, they constitute a great stakeholder in our decision-making. (Respondent 3, National AIDS and STI Control Program)

While discussing actors and stakeholders in the priority-setting process, many of the respondents highlighted the absence of the private sector during stakeholder engagements. One participant says:

The private sector [is missing] …the donor sources are dwindling, and government sources are already limited. So, we need to find new partners. We need to find corporate organizations / partnerships…we have been engaging them, but I think it could be more. (Respondent 3, National AIDS and STI Control Program)

#### Revision of priorities

In the event that priorities had to be revised, stakeholders were informed and engaged in the review process. The approaches described by respondents included re-evaluating priorities through compromise, negotiating extra funding, postponing the implementation for another time or reaching a consensus with stakeholders on what should be prioritised.

There are times you go to Ministry and say… this intervention is priority… they say ‘no no no… we don’t want to----- don’t prioritize ITN. We want to prioritize IRS’. Once they make such decision you have to come back and work towards that. (Respondent 1, Malaria Control program)

[We] go back to the people who gave us the money and tell them that… looking at what is happening, we may have to use this money for this. And that’s what we call reprogramming. All the reprogramed issues will have to be approved by the person who gave you the money. (Respondent 2, Malaria Control Program)

#### Power and influence

Participants described the influence stakeholders had in the priority setting. For some participants, the national institutions such as the MoH and its agencies were the most influential in the priority-setting process and implementation.

That would be the government sector broadly speaking. They have the most power because they eventually decide the decisions--- By that I mean the Ghana AIDS Commission, the Ministry of Health, the Ghana Health Service. (Respondent 8, WHO country rep)

According to other participants, development partners had the most power and influence on the priority-setting process.

it is their money so they decide which areas they want us to work in…there’s an intervention you want to put in, but if global fund does not see it as a priority we will not have funding for that… it’s the Global Fund. And they are influenced by what WHO says… (Respondent 7, National AIDS and STI Control Program)

Other respondents felt that despite funder’s influence, they did not dictate what priorities should be set.

Funders may have their interests and priorities, but they don’t impose things on countries, because at the end of the day the money comes to government, and government must agree that. I want to use the money on this…. (Respondent 5, TB Control program)

Stakeholders with the most power and influence could determine the duration of priority setting. The quotes below illustrates these instances:

If WHO says that… these drugs that are being used, we are changing the focus…every country is supposed to go by this. We quickly have to sit down and plan so that we can get all these stakeholders to come on board for us to review our guidelines and make sure that we disseminate it to the various treatment centers and the regions…So we don't actually have specific periods…we are been guided by this. (Respondent 6, Ghana AIDS Commission)

Previously we do it for three years but we have changed to four years to reflect the political mandate or the political term. We have note (sic) that most political parties have their own manifestos and promises that when they win power. You know the ministry works for the country and we are bound to implement those promises or manifestos. So, to ensure continuity of work…we have now taken a decision that the medium-term plan should coincide with the political term (Respondent 10, Ministry of Health)

### Transparency of the process

These responses captured participants’ descriptions about how they viewed the transparency of the priority-setting process. We found one sub-theme that illustrated this which is set out in more detail below.

#### Documentation and dissemination

According to participants, all actions taken to set priorities were documented, and dissemination of data was done through the sharing of reports, stakeholder meetings and making these documents available on the internet.

When you are done putting things together in meetings, you have it sent it back to them. They review it. And then you organize another stakeholder meetings for dissemination…. (Respondent 2, Malaria Control Program)

…the term that they would use is "country dialogue". So that whole documentation is also available on the Global Fund website and also guides the processes for the priority setting. (Respondent 8, WHO country rep).

### Contextual factors

Many respondents highlighted the contextual issues that acted as barriers to the priority-setting process. These included: (1) inadequate funding, (2) aligning priorities with funders and (3) interruptions in the priority-setting process.

#### Inadequate funding

For some participants, the lack of funding made the priority-setting and implementation process challenges. The first quote illustrates challenges with funding and engaging stakeholders in the priority-setting process. One participant says ‘*I'll say will be the funding for some of the processes because it can be an expensive process, because you need to consult so many stakeholders’ (Respondent 8, WHO country rep*).

I would say funding is number one--biggest. In fact, if there was money why do we have to go to priority setting? Because we know what is needed, and we can’t fund everything. (Respondent 1, Malaria Control program)

#### Aligning priorities with funders

Another challenge was navigating donor priorities and national/institutional priorities. In some instances, there were difficulties in getting donors to buy into the priorities that have been set for the country by national level institutions.

We sometimes complain that it’s too much. Because sometimes when the recommendations come, it is followed with associated changes in commodity access. For instance, if WHO says that we shouldn't use Nevirapin, because they've done the studies and realized that it doesn't give the same suppression compared to another medication, and they changed their guidelines, immediately, pharmaceutical manufacturing industry also changes. So we are all forced to go…. (Respondent 3, National AIDS and STI Control Program)

There are times when you have set priorities for the next five years or something, and you knew that hopefully some agencies will be giving you some money. And then they say that you have reprioritized their monies. So, you're not going to have your money as you thought. And then that also becomes a problem. (Respondent 2, Malaria Control Program)

Our development partners if you don’t manage them well trust me----- They also have their own agenda, they have their own operational strategies… and some of them, I mean, they can tell you that if they don't see these things [in your priorities], they cannot support you (Respondent 10, Ministry of Health)

#### Interruptions in the priority-setting process

Other participants described the processes of priority setting as a barrier. For one participant: ‘*that (priority setting) process can…in itself, affect the implementation…because it’s so many people, it slows down the process’ (Respondent 3, National AIDS and STI Control Programme*).

Another interruption to the process was the influence of politics in the priority-setting process as highlighted by this respondent.

You cannot take politics out of it. I mean, some of the decisions that you want take, and then the political environment may not support you. It could also be other interest groups like the CSOs also kicking against the decisions. (Respondent 10, Ministry of Health)

Other participants spoke to unforeseen circumstances that could affect the process negatively. Most notably, they spoke about the COVID-19 pandemic which was unexpected and disrupted the process.

Barriers like COVID came in unexpected… we had to move most of our trainings, uh, online… online can be good but you know, it has limitations. So… then some of the prioritized activities were delayed… distribution of nets to school children had to move because the schools are closed. (Respondent 4, TB Control program)

## Discussion

Based on the perspectives of key informants, we explored priority-setting approaches for three Global Fund-supported programmes in Ghana, namely, HIV/AIDS, TB and malaria. Our findings revealed that health needs were mainly identified from national or global mandates. This top-down approach in setting health programme needs is not uncommon for health systems in LMICs that rely on macro-level national decisions.^34^ Similar results have been found in Tanzania^35^ and Uganda.^36^

The engagement of the public, as well as those for whom interventions are meant to benefit, appears limited in the priority-setting process relative to other stakeholders such as policymakers, healthcare providers and academics.^3,34,37^ Setting programme health priorities that align with global or national priorities are useful in maintaining global health standards and outcomes^38^; however, there may be discrepancies between what individuals in the community and national representatives consider as health priorities.^39^ Limited participation of individuals from the general public is inconsistent with the stakeholder engagement components of some formal mechanisms that set goals to enhance public participation in health-system priority setting.^37,40^

We also identified limited participation from the private sector. The private health sector is a major actor in the provision of health services for many LMICs.^41^ The private sector is important for the supply and distribution of common goods for health as many developmental partners seek to reduce funding in LMICs.^42,43^ Although the private sector would be mainly motivated by profit-seeking market incentives, it is important to identify any interests that could align with public health actors to build health systems that are locally led and sustainable.^43–45^ Governments in LMICs who seek to expand private sector involvement in priority setting must consider critically the space that private-for-profit organisations can occupy in the health sector to safeguard health equity.^46,47^

Increasing participation of the general public and other stakeholders in priority setting requires active strategies that do not merely include giving the public a seat at the table. While this is an important step towards inclusion, stakeholders who have influence must attend to the power dynamics that may inhibit the inclusion of all voices in the priority-setting process.^48^ Strategies like those recommended by the International Association of Public Participation encourage stakeholder participation through consultation, involvement, collaboration and empowering stakeholders.^49,50^

We found that although the programme managers were involved in the priority-setting process by coordinating stakeholder engagements and generating evidence for priority setting, they appeared to have limited power in influencing the final decisions. Rather, the emphasis was on a central authority, either nationally (the MoH) or on global partners and funders as final decision-makers. Although some respondents were of the opinion that donor partners and funders did not dictate priorities, the line between autonomy in decision-making and coercion was hard to discern. The processes and outcomes of priority-setting are heavily dependent on how actors interact with varying distributions of power.^51,52^

Our findings suggest that actors within the process relied on multiple channels to communicate with each other. However, we found limited evidence on how these priorities were made publicly available to the general public. Although relevant documents may be accessed by the general public, institutional documents may contain technical language and other jargons that are not easily understood by individuals outside the field, further alienating the public from the process.^53^

Mechanisms for appeals and revisions appeared weak with no specific structure identified and no demonstration of the transparency of the process. Mechanisms for appeals and revisions appear to be limited in SSA more generally in priority setting.^35,53,54^ In the event that priorities had to be revised, respondents in our study indicated that the main stakeholders that were consulted were the funders. It is unclear if that implies that the main challenges that lead to revisions are financial or if there are implementation challenges which would require broader stakeholder consultations with, for example, beneficiaries. The absence of formal mechanisms for appeals and revisions may create more room for power disparities to influence the priority-setting process.

Similar to many LMICs, funding and capacity constraints were the main limitation to priority setting.^55^ We also identified political interference in the process as a major barrier. This arose when participants discussed selecting priorities to align with political promises and campaigns as well as donor interests. This finding is consistent with other studies that highlight the impact of the political context on the priority-setting process.^36,56,57^ Priority-setting processes that are based on evidence, transparency and engagement increase the public trust in outcomes and ensure the legitimacy of the priority-setting process. It also ensures that the appropriate health interventions are adopted to meet population health needs.^4^ Similar to other countries, the current pandemic also interrupted the regular schedules and activities of TB, HIV/AIDS and malaria programmes.^58^

We found that, generally, our respondents did not allude to a specific framework or explicit approach to priority setting. Even among those who relied on their own internally generated data and surveillance, we did not identify an explicit approach that guided the generation and analysis of such data. Although we did not specifically ask participants to speak to an explicit mechanism for priority setting, we expected that it might appear in their responses on their approaches to priority setting.

Studies have demonstrated how approaches like MCDA and HTA can be used for effective and transparent allocation of scarce health resources in Ghana.^1,59,60^ Addo *et al*,^56,61^ however, found that knowledge of HTA among health policy actors was very limited, and further training is required before HTA can be implemented successfully. Despite these concerns Hollingworth and colleagues^62,63^ demonstrate the potential of HTA as a priority-setting mechanism in Ghana for effective resource allocation.

### Recommendations for policy

The mechanism for priority setting, in particular the use of explicit approaches like HTA, seems limited in three programmes linked to GFATM funding in Ghana. The MoH and its agencies will benefit from regular training in these explicit approaches for more transparent and precise decision-making on health priorities.

In the use of the current decision-making process for health, the MoH and its agencies should encourage and increase the engagement of individuals from the general public, the private sector and civil society organisations (CSOs) in priority-setting processes. Emerging challenges in funding can sometimes be supported by the private sector once a business case is presented to them indicating the benefits of investment in these three disease programmes.^64^ CSOs can also play advocating roles to support current and emerging issues.

Ghana’s lower middle-income status has fiscal, financial and priority-setting implications. While it seems development partners play a dominant role in decisions for the three diseases, the seemingly gradual withdrawal of funding will have implications for their participation in decision-making (priority setting included). This implies that the dynamics are gradually changing and the MOH and relevant agencies for the three diseases need to be adjusted and dominated in these processes.

### Limitations and implications for future studies

Our qualitative methodology limits the generalisability of this study. Our sample consisting of mostly male participants also limited our ability to explore gender dynamics and considerations inherent in the priority-setting approaches for the three disease programmes. We did not ask respondents to speak directly to frameworks or explicit mechanisms used in priority setting. As a result, we may have missed some vital information.^18,61^ Our findings suggest that the key informants we sampled were not in a position to directly select priorities, rather to inform or contribute to priority setting. Future studies should consider sampling higher-level decision-makers directly involved in the priority-setting process to determine what framework or explicit approaches are used. Future studies should also consider finding out what these people perceive their role to be in the priority-setting process. For this study, we did not specifically seek to explore differences in priority-setting approaches for the three disease programmes. As such, we may have missed vital information specific to each disease programme. Future studies should consider exploring any such differences.

## Conclusion

The study revealed that main actors in decision-making or priority setting for the three diseases were ministries and agencies, development partners, research institutions, committees and working groups. The priority-setting approaches rely on national health strategies and mandates, development partners and global mandates, as well as country generated data and surveillance information. We found, overall, that there was very little involvement of general members of the public in the priority-setting process. Stakeholder involvement in the process was dominated by actors from MoH and health partners (mainly the WHO and Global Fund); these groups had the most influence in the process. Our findings suggest that transparency of the process may be more evident to actors within the process compared to those not directly involved. Mechanisms for appeals and revisions also appeared weak. Contextual factors identified related mainly to barriers in the priority-setting process, which included inadequate funding, aligning priorities with funders and interruptions in the priority-setting process. The use of explicit mechanisms such as HTA and related approaches appears limited when setting priorities in GFATM-supported programmes in Ghana. From this study, one main implication for policymakers and practitioners is that, while reliance on national and global health strategies as well as country-generated data is a feasible process for decision-making, it is important to consider using recognised priority-setting frameworks and explicit approaches.

## Supplementary material

10.1136/bmjph-2024-001097 online supplemental file 1

10.1136/bmjph-2024-001097 online supplemental file 2

## Data Availability

Data are available upon reasonable request.
