# Supplementary material for: What are the priority-setting approaches for HIV/AIDS, TB and malaria programmes in Ghana? A qualitative perspective from key informants
Source: BMJ Public Health. 2024 Dec 12;2(2):e001097. doi: 10.1136/bmjph-2024-001097 (PMC12580987; doi:10.1136/bmjph-2024-001097)
Supplement: Supplementary file 1 [file bmjph-2-2-s001.pdf]

**Appendix 1**

**Interview guide**

1. Current position, role and number of years of service
2. What is your understanding of priority setting for your program?
3. Please describe the organization's decision-making process?
4. Can you identify (list) actors involved and their roles in decision making in priority setting?
5. What is the extent of actor's involvement in the priority setting process?
6. Which actors have the most influence in priority setting decision making and why?
7. Which actors could have been engaged that were not and why?
8. Describe the extent of documentation and dissemination of the priority setting process.
9. Were there mechanisms or avenues for revision of priority setting decisions? How are these mechanisms implemented?
10. What contextual factors (barriers and facilitators) influence the priority setting process, how and why?
