# Supplementary material for: What are the priority-setting approaches for HIV/AIDS, TB and malaria programmes in Ghana? A qualitative perspective from key informants
Source: BMJ Public Health. 2024 Dec 12;2(2):e001097. doi: 10.1136/bmjph-2024-001097 (PMC12580987; doi:10.1136/bmjph-2024-001097)
Supplement: Supplementary file 2 [file bmjph-2-2-s002.pdf]

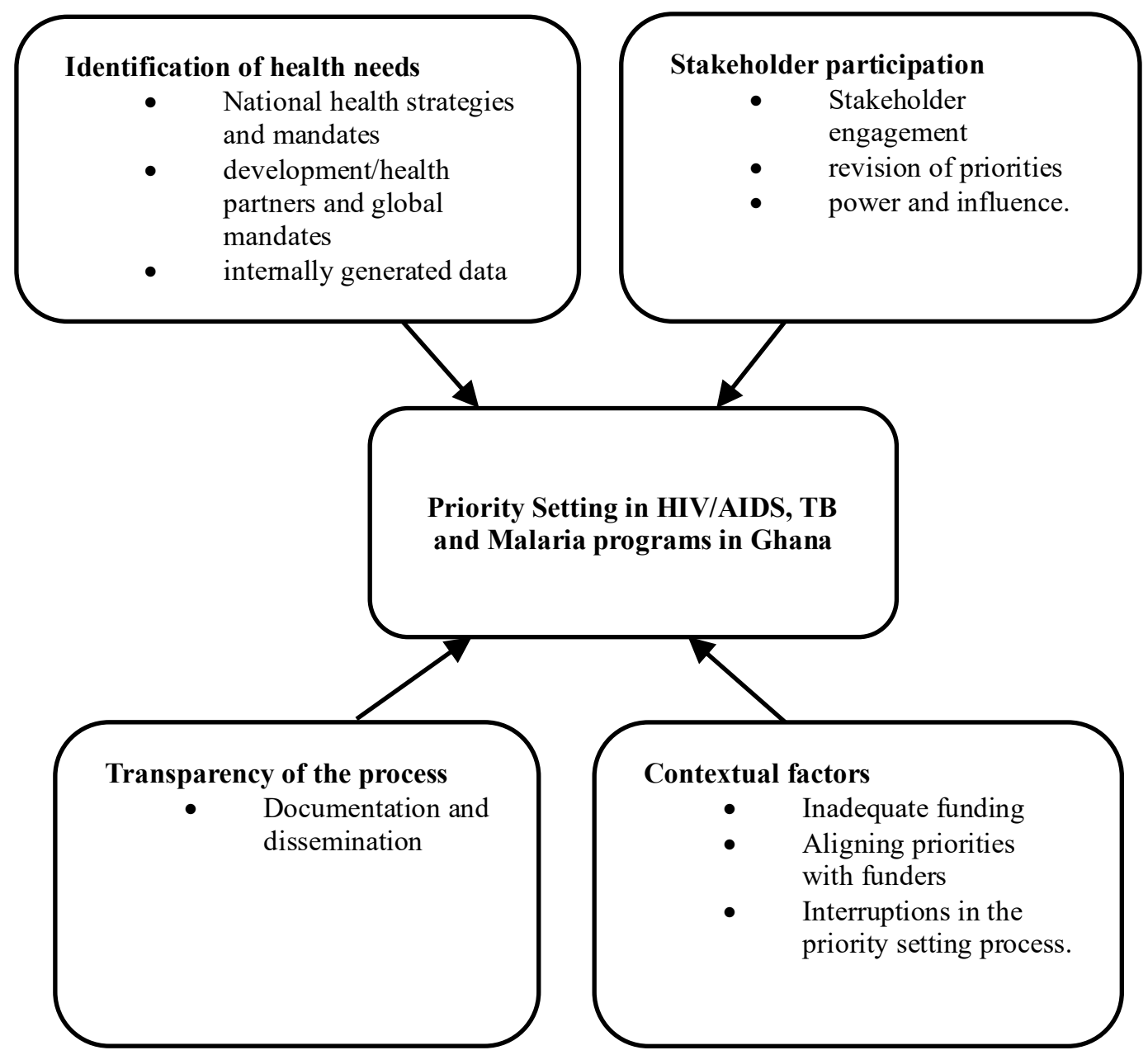

Fig 1. Priority setting approaches for HIV/AIDS, TB and Malaria programs in Ghana
